# Oviposition by *Spodoptera exigua* on *Solanum dulcamara* Alters the Plant’s Response to Herbivory and Impairs Larval Performance

**DOI:** 10.3390/ijms19124008

**Published:** 2018-12-12

**Authors:** Daniel Geuss, Tobias Lortzing, Jens Schwachtje, Joachim Kopka, Anke Steppuhn

**Affiliations:** 1 Molecular Ecology, Dahlem Centre of Plant Sciences, Institute of Biology/Freie Universität Berlin, Albrecht-Thaer Weg 6, 14195, Berlin, Germany; d.geuss@fu-berlin.de (D.G.); tobias.lortzing@fu-berlin.de (T.L.); 2 Applied Metabolome Analysis, Max-Planck-Institute for Molecular Plant Physiology, Am Mühlenberg 1, 14476 Potsdam-Golm, Germany; schw8je@gmail.com (J.S.); kopka@mpimp-golm.mpg.de (J.K.)

**Keywords:** Plant defense, primary and secondary metabolism, defense priming, insect eggs, protease inhibitors, herbivory, transcriptomics, metabolomics, microarray

## Abstract

Plant resistance traits against insect herbivores are extremely plastic. Plants respond not only to the herbivory itself, but also to oviposition by herbivorous insects. How prior oviposition affects plant responses to larval herbivory is largely unknown. Combining bioassays and defense protein activity assays with microarray analyses and metabolite profiling, we investigated the impact of preceding oviposition on the interaction of *Solanum dulcamara* with the generalist lepidopteran herbivore *Spodoptera exigua* at the levels of the plant’s resistance, transcriptome and metabolome. We found that oviposition increased plant resistance to the subsequent feeding larvae. While constitutive and feeding-induced levels of defensive protease inhibitor activity remained unaffected, pre-exposure to eggs altered *S. dulcamara*’s transcriptional and metabolic response to larval feeding in leaves local and systemic to oviposition. In particular, genes involved in phenylpropanoid metabolism were more strongly expressed in previously oviposited plants, which was reflected by reciprocal changes of primary metabolites upstream and within these pathways. Our data highlight that plants integrate signals from non-threatening life stages of their natural enemies to optimize their response when they become actually attacked. The observed transcriptional and metabolic reshaping of *S. dulcamara*’s response to *S. exigua* herbivory suggests a role of phenylpropanoids in oviposition-primed plant resistance.

## 1. Introduction

Plants have evolved numerous traits to resist or prevent herbivory. These can be constitutively expressed or induced upon herbivore attack [1]. Inducible defense traits allow plants to restrict their investments into resistance to situations of herbivore attack and to tailor their response specific to the attacker. A drawback of inducible defense is the risk of substantial damage during the time required to establish effective resistance, which can take up to several days [1]. However, not only herbivory itself can increase plant resistance to herbivores but also stimuli that are predictive of an upcoming attack. For example, plants exposed to volatiles released from adjacent herbivore-attacked plants can enhance their own inducible defenses [2,3,4]. 

Oviposition by insect herbivores on a host plant poses a particularly high risk of future herbivory and can enable plants to respond to the attacker even before actual damage occurs [5]. Some of those responses can reduce egg survival, either directly, such as by the production of ovicidal substances, [6,7] or indirectly, such as by the activation of chemical signals to attract egg parasitoids [8,9]. Furthermore, insect oviposition can facilitate increased plant resistance to subsequently feeding larvae which is reflected by their reduced performance on previously egg laden plants [10,11,12,13,14], but how this is mediated is largely unknown.

On the one hand, oviposition may induce plant defense traits against the feeding larvae. On the other hand, plant responses to oviposition may alter how plants respond to the feeding larvae and thereby increase plant resistance. An example of the latter is the enhanced inducibility of two anti-herbivore defense traits upon larval feeding when *Nicotiana attenuata* plants were previously oviposited by *Spodoptera exigua* [11]. Larvae of this noctuid herbivore suffer reduced performance on previously oviposited plants, which showed an increased induction of protease inhibitor (PI) activity and content of caffeoylputrescine, a phenylpropanoid-polyamine conjugate. Plants that are deficient for both defense traits due to gene silencing of the transcription factor *NaMyb8* [15,16] are also incapable of improving their resistance against *S. exigua* larvae after oviposition [11]. Thus, when *N. attenuata* is oviposited, it enhances its feeding-induced defense as a resistance strategy. Also, tomato plants that had been oviposited by a noctuid moth increase their inducibility of PI gene expression and of the phytohormone jasmonic acid (JA) [17], the key regulator of plant defense responses to chewing herbivores [18]. 

Contrasting results were obtained for egg-mediated effects in *Arabidopsis thaliana*. The expression of feeding-induced defense genes is attenuated and larval performance of *Spodoptera littoralis* is increased after a treatment with egg-extract of *Pieris brassicae* [19]. Both effects require intact biosynthesis of salicylic acid (SA), a phytohormone that is induced by oviposition and that can antagonize JA signaling [19,20,21]. Accordingly, the reduced resistance of *A. thaliana* to *S. littoralis* after egg-extract treatment is attributed to a negative cross-talk between egg-induced SA and feeding-induced JA signaling. Yet, performance of *P. brassicae* larvae on *A. thaliana* can also be reduced due to its oviposition on the plant [13,22]. Similarly, pre-treatment of *Brassica nigra* plants with *P. brassicae* egg-extract reduced larval performance of *P. brassicae* larvae, despite the induction of SA signaling and a reduced induction of JA-mediated gene expression [23]. Hence, the effect of insect oviposition on plant resistance to feeding larvae seems to vary for different insect-plant interactions.

Few studies used transcriptome analyses to survey plant responses to insect eggs and they determined substantial transcriptional changes in *A. thaliana*, *B. nigra* and *Solanum dulcamara* [6,23,24,25]. In response to moth oviposition, all three species showed an up-regulation of genes involved in plant defense responses to pathogens, in responses to oxidative stress and in phenylpropanoid metabolism. Other than in these plant species, oviposition by the elm leaf beetle *Xanthogaleruca luteola* inflicts damage and regulates the plant’s transcriptome more similarly to the JA-mediated wound response [26].

Recent studies also evaluated how the feeding-induced plant transcriptome is affected by insect eggs. The transcriptional response of *Ulmus minor* to feeding *X. luteola* larvae was faster when the plants were oviposited one week earlier but not qualitatively altered [26]. The latter was found for the transcriptomic response of *A. thaliana* to herbivory by *P. brassicae* larvae, which was altered in more than 200 genes due to a previous oviposition by the same species [22]. In contrast to that, pre-treatment of *B. nigra* plants with *P. brassicae* egg-extract did not or only marginally affected the plant’s transcriptional response to subsequent *P. brassicae* herbivory [23]. Also, when generalist *S. exigua* or specialist *Manduca sexta* larvae were feeding on *N. attenuata* that were oviposited by conspecific moths, only marginal differences were found one day after onset of larval feeding [27]. Yet, the feeding-induced transcriptional response of the plant to both species differed tremendously and oviposition by *S. exigua* shifted the species-specific imprint of *M. sexta* larvae towards the *S. exigua*-specific imprint and vice versa [27]. Together, these studies underline that the oviposition by an insect herbivore is an important but largely overlooked aspect of herbivore-plant interactions that affects how plants respond to the herbivorous stage with consequences for the plant’s resistance and the insect performance. However, our knowledge on the effects of insect oviposition on the plant’s response to the feeding larvae is still fragmentary. Thus, more studies are required to identify general patterns of oviposition-mediated modulation of plant responses to herbivory but also those aspects that are very specific to the investigated insect–plant interaction. 

We investigated how oviposition by the generalist herbivore *S. exigua* on *S. dulcamara*, a wild model species that is closely related to crop species such as tomato and potato, affects the plant’s interaction with feeding *S. exigua* larvae. As we had shown previously, *S. dulcamara* responds to oviposition by *S. exigua* with an effective direct defense against the eggs through reactive oxygen species produced in tissue beneath the eggs [6]. Therefore, it is a promising model system to explore whether and how oviposition on the plant affects the performance of later feeding larvae. We found that a preceding oviposition also increased plant resistance to *S. exigua* larvae in *S. dulcamara*. Because this species also responds to herbivory with the production of defensive PI activity [28,29], we then examined whether feeding-inducible PI activity is altered in oviposited plants as it is in other Solanaceae [11,17]. Furthermore, we examined how oviposition affects the plant’s phytohormonal, transcriptional and metabolic responses to larval feeding. One day after the onset of larval feeding, we found no effects of a preceding oviposition on phytohormone levels but on *S. dulcamara*’s feeding-induced transcriptome and metabolome. At both levels, an altered induction of phenylpropanoids was indicated, which we discuss together with recent studies in other plant species, as a general target of oviposition-priming in plant metabolism.

## 2. Results

### 2.1. Moth Oviposition on S. dulcamara Impairs Performance of Subsequently Feeding Larvae

**Figure 1:**
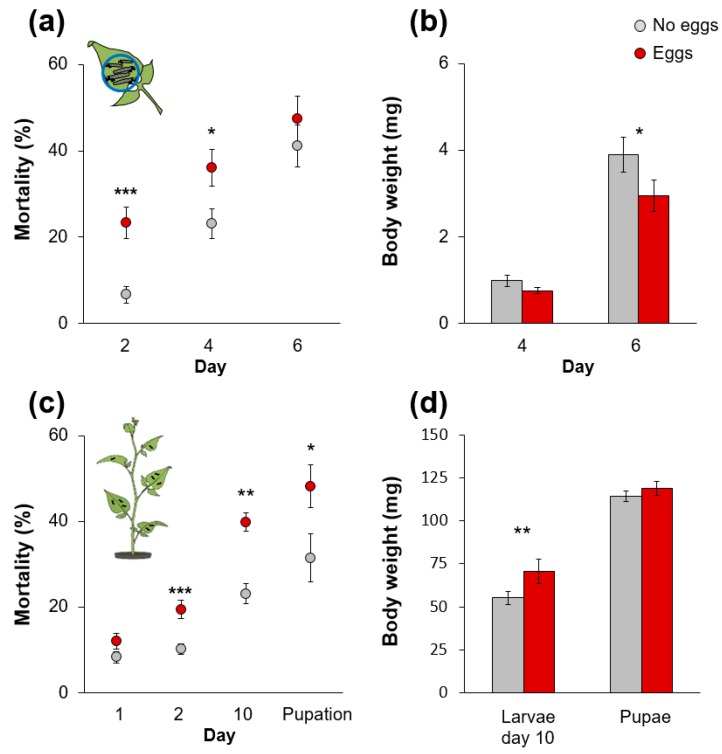
Larval performance of *S. exigua* on oviposited *S. dulcamara* plants. (**a**,**c**) Mortality and (**b**,**d**) body weight (mean ± SE) of *S. exigua* larvae on previously oviposited (Eggs) and non-oviposited plants (No eggs). Larvae were either (**a**–**b**) confined on a single leaf which was changed every second day (*N* = 23–25) or (**c**–**d**) were allowed to freely move on the whole plant after two days on defined leaf positions (*N* = 9). Asterisks indicate significant differences according to generalized linear mixed models (**a**,**c**) or linear mixed models (**b**,**d**) at *p* < 0.05/0.01/0.001 (*/**/***).

We used two different setups to test whether oviposition by *S. exigua* on *S. dulcamara* would affect the performance of subsequently feeding *S. exigua* larvae. In the first setup, we confined six larvae on a single leaf. Within the first two days, larvae on oviposited plants suffered a three-fold higher mortality than larvae on non-oviposited plants. Thereafter, larval mortality also vastly increased on non-oviposited plants up to about 40% at day six, which was similar to the mortality on oviposited plants (Figure 1a). However, the larvae surviving until day six on non-oviposited plants gained significantly more weight than those on oviposited plants (Figure 1b). 

**Figure A1:**
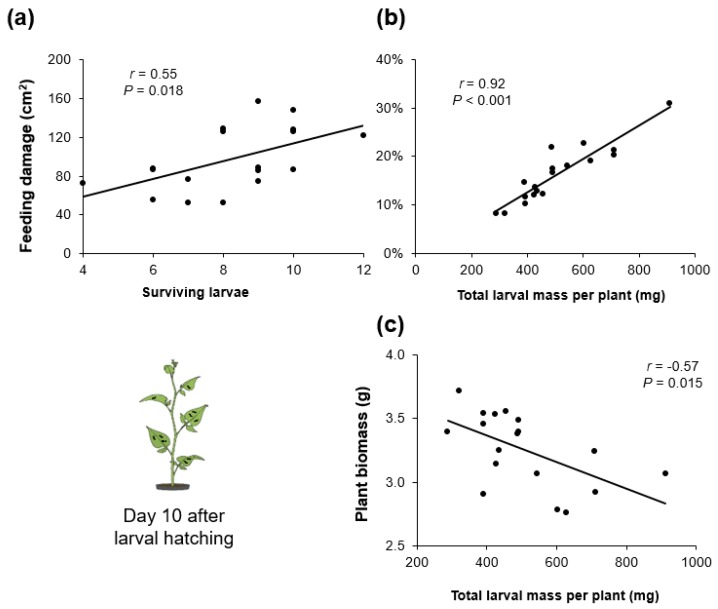
Feeding damage and biomass of *S. dulcamara* plants on which 12 *S. exigua* neonates were released. Correlations of (**a**) feeding damage in cm^2^ with the number of larvae that survived on each plant until day 10 as well as of (**b**) feeding damage (in % damage of the total leaf area) and (**c**) plant biomass with the total larval mass (sum of all individual larval weights) per plant (*N* = 18; half of the plants were previously oviposited). Correlation coefficients (r) and *p*-values according to Pearson’s product-moment correlation are depicted.

In a second, more natural setup we released larvae onto the whole plant after two days of feeding on the oviposited and the next-youngest leaves. In this setup, the higher mortality of larvae on oviposited plants persisted from day two until pupation (Figure 1c). The fewer larvae surviving on oviposited plants until day 10 weighed more than larvae on non-oviposited plants but this difference was not retained to the pupal stage (Figure 1d). The amount of feeding damage on the plants correlated positively with the number of larvae that survived until day 10 (Pearson correlation: r = 0.55, *p* = 0.018; Appendix A: Figure A1). Similarly, both the proportion of leaf area consumed by larvae and plant biomass correlated most strongly (r = 0.92, *p* < 0.001 and r = -0.57, *p* = 0.015, respectively) with the sum of all larval weights on a plant at day 10. However, likely due to the opposing effects of prior oviposition on larval survival and weight gain, neither feeding damage nor plant biomass differed between oviposited and non-oviposited plants.

### 2.2. Feeding-Induced PI Activity in S. dulcamara Is Not Altered by Prior Oviposition 

**Figure 2:**
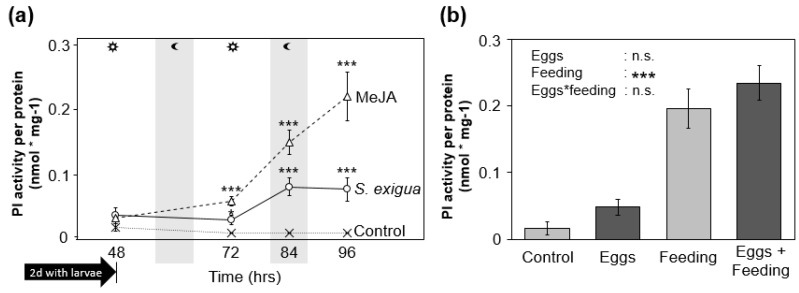
Protease inhibitor (PI) activity in *S. dulcamara* leaves (mean ± SE) after larval feeding and/or oviposition. (**a**) Inducibility of PI activity at different time points after a two-day feeding period by a single third-instar *S. exigua* larva *(N* = 9) in comparison to plants treated with 150 µg methyl jasmonate (MeJA) in lanolin (*N* = 7) at the beginning of the feeding period and to untreated control plants (*N* = 6–8). (**b**) PI activity of control plants and previously oviposited plants (Eggs) three days after 20 *S. exigua* neonates were released on an additional set of oviposited (Eggs + Feeding) and non-oviposited plants (Feeding) (*N* = 6 for plants with and *N* = 3 for plants without feeding). Larvae were allowed to feed for three days. Asterisks indicate (**a**) significant differences from control plants at each time point according to Welsh’s *t*-tests or (**b**) significant effects of the factors (Eggs and Feeding) in linear mixed models at *p* < 0.001 (***; n.s.: not significant).

Similar to other plants, trypsin PI activity is inducible in *S. dulcamara* in response to herbivory and methyl jasmonate (MeJA) application (Figure 2a). It remained at constitutive levels in leaves that had been fed by a single *S. exigua* larva for two days but significantly increased one day later and even further within the next day. In the full-factorial experiment, PI activity was strongly increased in leaves on which initially 20 *S. exigua* neonates were released and could feed for three days, but this induction was not altered due to previous oviposition, nor did oviposition per se affect PI activity (Figure 2b).

### 2.3. S. dulcamara’s Phytohormonal Response to Larval Feeding and Oviposition 

**Figure 3:**
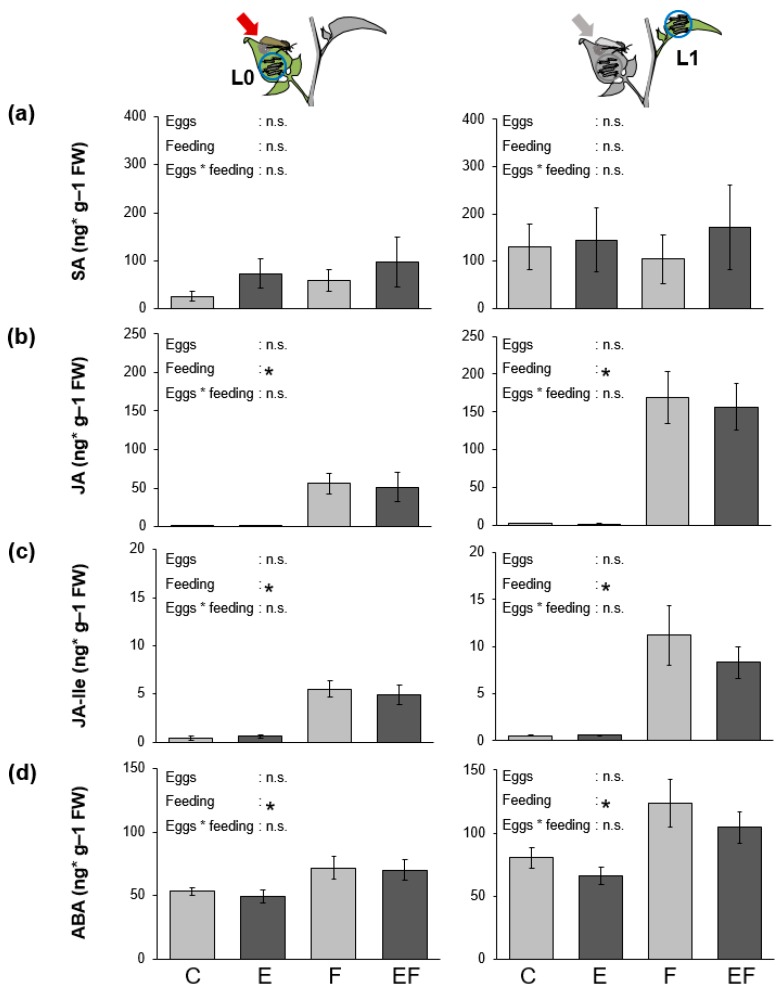
Feeding induction of phytohormones in *S. dulcamara* is not altered by oviposition. Levels of (**a**) salicylic acid (SA), (**b**) jasmonic acid (JA), (**c**) JA-isoleucine (JA-Ile) and (**d**) abscisic acid (ABA) (mean ± SE, *N* = 9) in *S. dulcamara* leaves local (left panel: L0-leaf) or systemic (right panel: L1-leaf) to the leaf subjected to oviposition (marked with arrows). Plants were exposed to *S. exigua* herbivory (Feeding: F), herbivory with prior oviposition (Eggs + feeding: EF), oviposition alone (Eggs: E) or were left untreated (Control: C). Asterisks indicate significant differences according to linear mixed models (LMMs) at *p* < 0.05.

In another full-factorial experiment, we analyzed the levels of phytohormones in untreated control plants (C), previously oviposited plants (E), plants fed upon by *S. exigua* larvae (F) and previously oviposited plants subsequently fed upon by larvae (EF). At the analyzed time points, either one day (L0-leaf) or two days (L1-leaf) after the egg removal, we found no differences between C- and E-plants. Larval feeding for 24 h increased levels of JA, JA-isoleucine (JA-Ile), and abscisic acid (ABA) in both leaf positions, the L0-leaf that was previously exposed to the eggs and the next-youngest L1-leaf that was fed upon one day later (Figure 3). However, neither levels of SA, JA, JA-Ile or ABA differed between EF-plants and F-plants.

### 2.4. Oviposition Modifies S. dulcamara’s Transcriptional Response to Larval Feeding 

**Figure 4:**
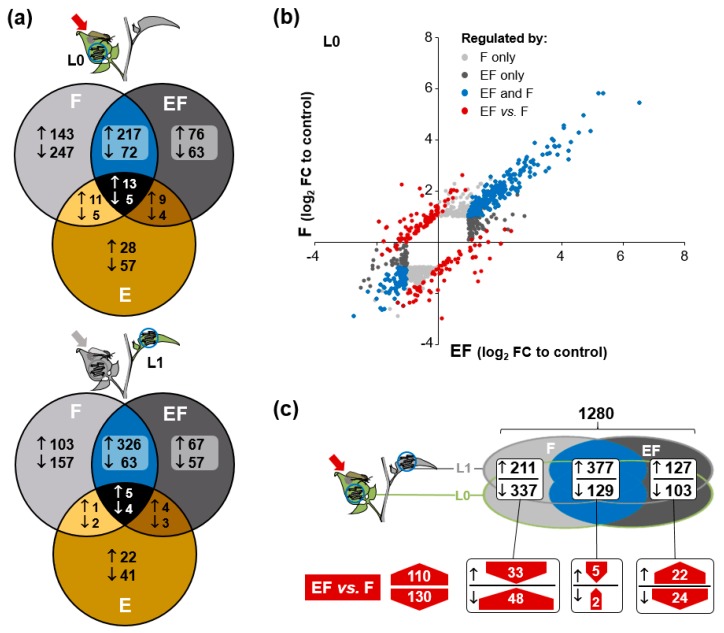
Transcriptional response to larval feeding on oviposited and non-oviposited plants. (**a**) The gene sets of the microarray analyses (*N* = 3) of the previously oviposited leaf or the same leaf position of non-oviposited plants (L0) as well as the next-youngest leaf (L1). Numbers represent genes up (↑)- and down (↓)-regulated (log_2_-fold change (FC) > 1, *p* < 0.05) by oviposition (Eggs: E) or larval feeding of *S. exigua* for 24 h on previously oviposited (Eggs + feeding: EF) and non-oviposited (Feeding: F) plants relative to untreated control plants. (**b**) Gene expression changes (log_2_-FC) in the L0-leaf in F- and EF-plants are plotted against each other and genes only regulated in either F- or EF-plants are displayed in light or dark grey, genes regulated by both treatments are shown in blue, and genes with a differential expression between F- and EF-plants are marked in red (see Appendix A: Figure A2 for L1 leaf). (**c**) The analyses of both leaf positions are combined as illustrated in the Venn plot to depict the overlaps between feeding-responsive genes (relative to control plants) and genes differentially expressed between EF- and F-plants (red arrows are oriented upwards if these genes are higher and downwards if these are lower expressed in EF- than in F-plants).

To further investigate what plant responses could be related to the altered larval performance on oviposited plants, we performed a non-targeted transcriptome analysis of *S. dulcamara*’s transcriptional response to larval feeding in plants with and without previous oviposition. Using a custom microarray for *S. dulcamara* we analyzed two sequentially attacked leaf positions, local (L0) and systemic (L1), to prior oviposition. In the L0-leaf, 950 genes (and 855 genes in the L1-leaf) were differentially expressed in E-, F- and EF-plants relative to control plants (Figure 4a, see Supplementary material: Table S1 for all genes differentially regulated between any of the treatments in both leaves). More than 90% of these genes were altered in plants that received larval feeding (F- and EF-plants) and only 5% and 2.5% of these genes were among the 132 and 82 genes that were differentially expressed between E- and control plants in the L0- and L1-leaf (marked in ocher in Figure 4a), respectively. Thus, one day after egg-exposure, oviposition per se left a marginal transcriptional imprint barely overlapping with feeding responsive transcripts.

**Figure A2:**
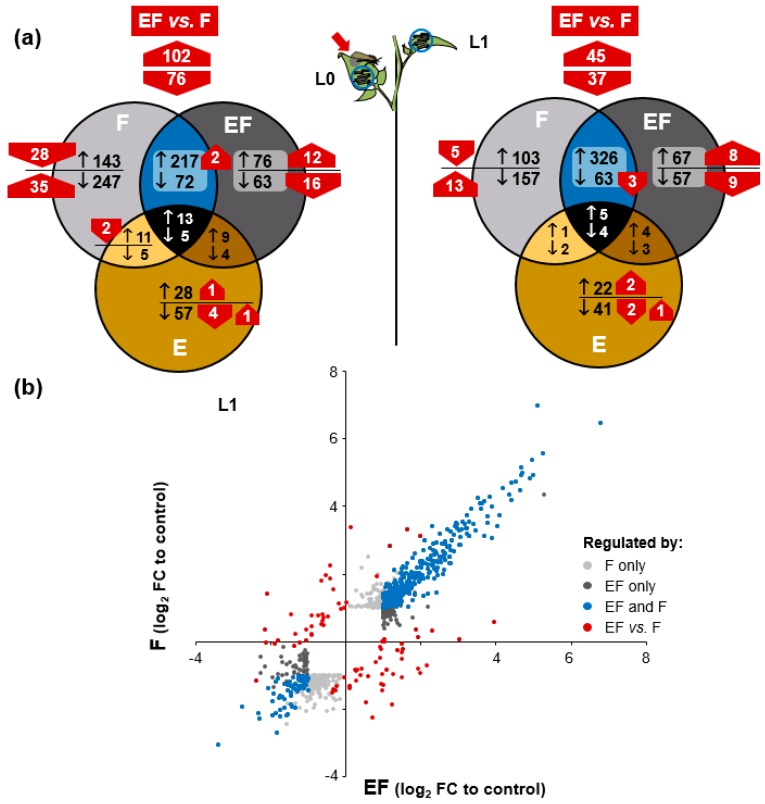
*S. dulcamara*’s transcriptional response to larval feeding on oviposited and non-oviposited plants. Microarray analyses (*N* = 3) of the L0-leaf position previously exposed to oviposition by *S. exigua* as well as the next younger L1-leaf. (**a**) Venn plots with the numbers of up (↑)- and down (↓)- regulated genes (log_2_-fold change (FC) > 1, *p* < 0.05) in response to oviposition (Eggs: E), larval feeding (Feeding: F) and the combination of both (Eggs + feeding: EF) relative to untreated control plants. The red arrows demark the number of genes differentially expressed between F- and EF-plants and those in the Venn plots how many of them overlapped with the genes differentially expressed between treated and control plants. (**b**) Gene expression changes (log_2_-FC) in the L1-leaf in F- and EF-plants relative to control plants are plotted against each other. Genes only regulated in either F- or EF-plants are displayed in light or dark grey while genes regulated by both treatments are shown in blue and genes with a differential expression in F- and EF-plants are marked in red (see Figure 4b for the L0-leaf).

The response to larval feeding overlapped between F- and EF-plants to only one third in the L0-leaf and about half in the L1-leaf. This common response of F- and EF-plants (marked in blue in Figure 4a) was dominated by up-regulated genes in both leaf positions and includes the strongest regulated genes in response to feeding (Figure 4b, Appendix A: Figure A2). About 45% of the feeding-responsive genes in the L0-leaf were exclusively altered in F-plants (light grey). These genes were dominated by down-regulated genes. This was similar in the L1-leaf in which one third of the feeding-responsive genes were exclusively altered in F-plants. In both leaf positions, around 16% were exclusively altered in EF-plants (dark grey). The ratio between up- and down-regulation was relatively balanced in the genes specifically regulated in EF-plants. 

**Figure A3:**
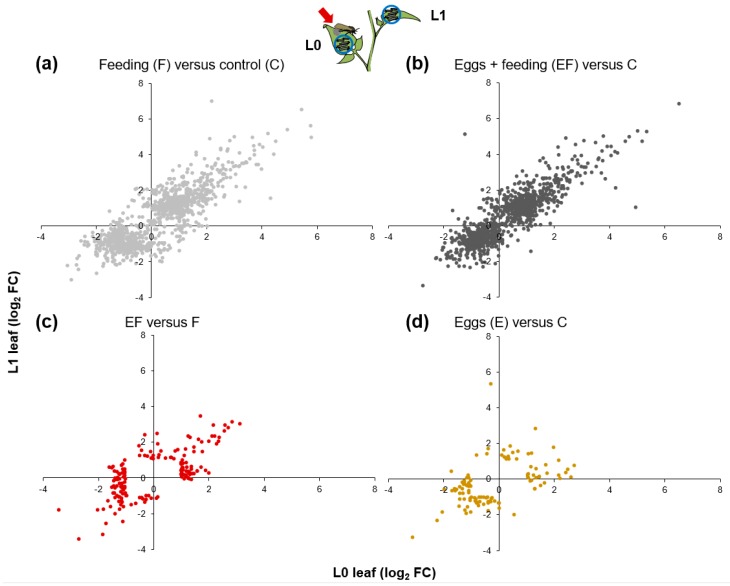
Log-log-plots comparing *S. dulcamara*’s transcriptional response to larval feeding and oviposition by *S. exigua* in both analyzed leaves by microarrays (*N* = 3). The plants were either untreated (Control: C), previously oviposited (Eggs: E), fed larvae (Feeding: F) or previously oviposited and fed by larvae (Eggs + feeding: EF). The leaf L0 was previously exposed to oviposition by *S. exigua* and subsequently fed by larvae for one day while the next younger L1 leaf was fed by the larvae 1 day later. (**a**) Log_2_-fold changes (FC) relative to untreated control (C) plants in response to feeding by *S. exigua* larvae for 24 h in the L0-leaf (x-axis) are plotted against those in the L1-leaf (y-axis) for all genes regulated in F-plants in at least one of the two leaves. Similar log-log-plots are displayed for all genes differentially regulated (**b**) in EF-plants relative to C-plants, (**c**) in EF-plants relative to F-plants and (**d**) in E-plants relative to C-plants.

The large fraction of feeding-responsive genes that were not shared between F- and EF-plants suggests an altered transcriptional response to the larvae due to the preceding oviposition. To explore this possibility, we directly compared the transcriptomes of F- and EF-plants to each other. We found 178 genes in the L0-leaf and 82 genes in the L1-leaf that were differentially expressed in F- and EF-plants. Many of these genes showed an inverse regulation in F- and EF-plants relative to control plants (red in Figure 4b and Appendix A: Figure A2) and more than half of these were among the feeding-responsive genes in both leaf positions (Appendix A: Figure A2). Gene expression in both leaf positions correlated well and showed very similar regulation patterns for the treatments (Appendix A: Figure A2 and Figure A3) and we summarized these multiple comparisons of the analyses for both leaf positions (Figure 4c). Among the 1280 genes that were altered relative to control plants in F- and EF-plants in both or either of the two leaf positions, slightly more than 10% (131 genes) were differentially expressed between F- and EF-plants when directly compared. These account for 65% of the genes with a higher expression and almost half of the genes with a lower expression in EF-plants than in F-plants, while just 15% of the genes exclusively altered in F-plants and 20% of the genes exclusively altered in EF-plants were significantly different between F- and EF-plants. Thus, this analysis could substantiate an altered transcriptional response to larval feeding by prior oviposition for about a fifth of the genes that are only altered in one of the feeding treatments and suggests that there are also transcriptional changes among genes that show less distinct expression differences compared to control plants and among those that are changed in a similar direction in F- and EF-plants. 

**Figure A4:**
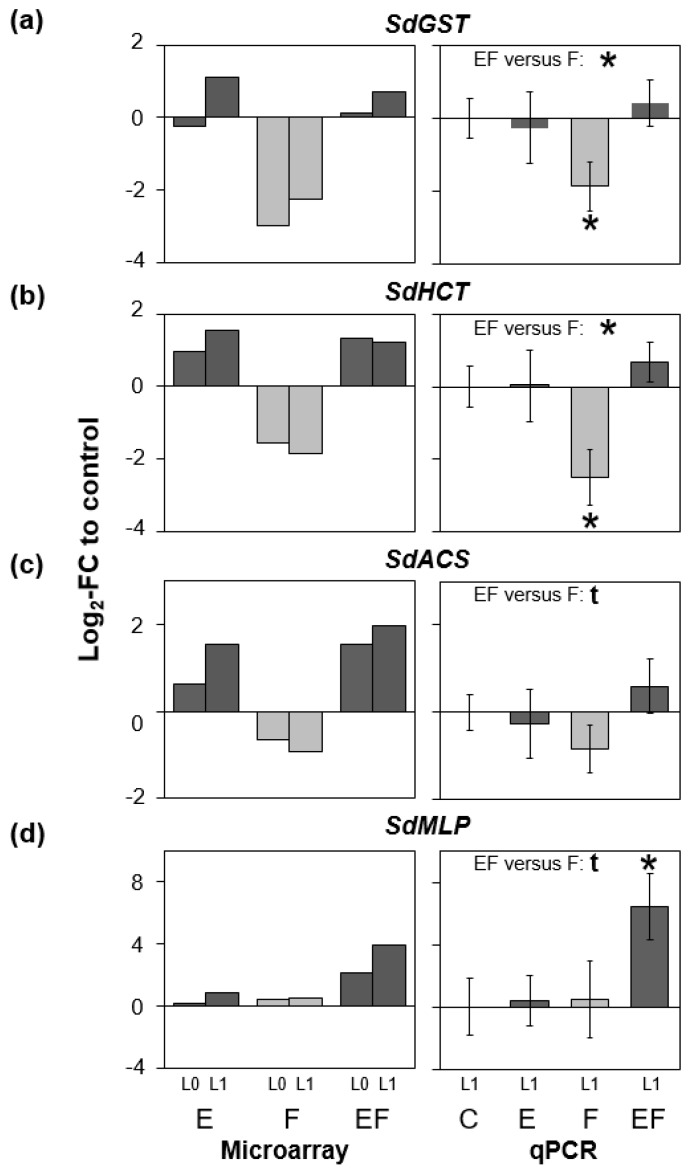
RT-qPCR validation for selected genes on the microarray. Transcripts were determined in RNA from leaf tissue of *S. dulcamara* plants that were either untreated (Control: C), previously oviposited (Eggs: E), fed by *S. exigua* larvae (Feeding: F) or fed and previously oviposited (Eggs + feeding: EF). Two leaf positions consecutively exposed to 24 h of larval feeding were analyzed; the leaf position subjected to oviposition (L0) and the next younger leaf (L1). Four genes that were differentially expressed between F- and EF-plants in a microarray analysis of RNA pools (N = 3, left panel) were selected: (**a**) *glutathione S-transferase* (*GST*), (**b**) *hydroxycinnamoyl CoA quinate transferase* (*HQT*), (**c**) *anthocyanidin synthase* (*ANS*) and (**d**) *major latex-like protein* (*MLP*) and subjected to qPCR (right panel) on the RNA from L1-leaves of individual plants (*N* = 8-9). Log_2_-fold changes (FC) (mean ± SE) in transcript accumulation relative to C-plants after normalization to the reference gene *elongation factor 1* (*ELF1*). Asterisks indicate significant differences (*p* < 0.05) and “t” a trend (*p* < 0.1) according to Welch’s *t*-tests.

The largest number (48) of feeding-regulated genes that were differentially expressed between F- and EF-plants were down-regulated in response to larval feeding but their expression in EF-plants was not different from control plants (Figure 4c). Among them were genes of the core phenylpropanoid pathway, such as *hydroxycinnamoyl-CoA quinate transferases* (*HQT*), the oxidative stress-related genes e.g., *glutathione S-transferase* (*GST*), and signaling-related genes e.g., *ethylene responsive transcription factors*. We confirmed by qPCR the induction patterns of two of those genes (*HQT*, *GST*), of a gene up-regulated solely in EF-plants (*major latex-like protein*) and of a gene differentially expressed between EF- and F-plants but not in relation to C-plants (*anthocyanidin synthase*) (Appendix A: Figure A4).

### 2.5. Genes of Phenylpropanoid-Related Pathways Were More Strongly Expressed in Previously Oviposited Plants 

**Figure A5:**
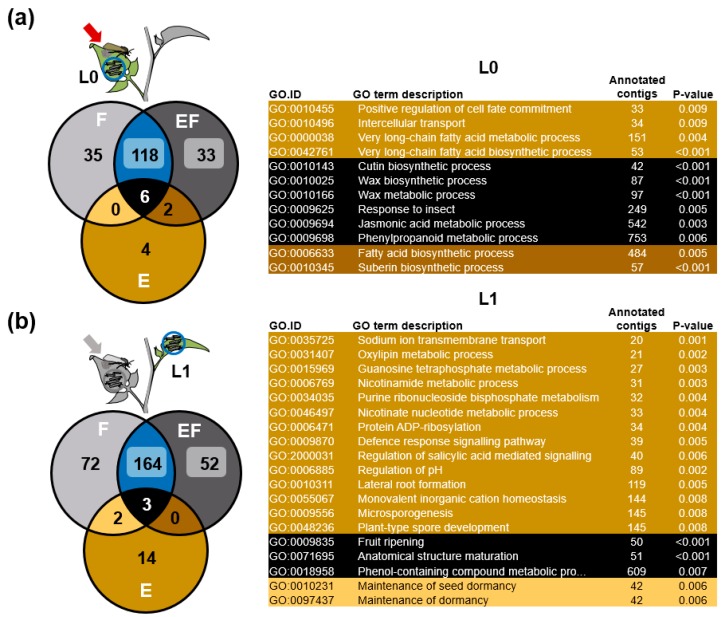
GO-enrichment for genes differentially expressed relative to control *S. dulcamara* plants in response to *S. exigua* oviposition (Eggs: E), larval feeding (Feeding: F) and the combination of both (Eggs + feeding: EF). Differentially expressed (log_2_-fold change > 1, *p* < 0.05) genes were analyzed for significantly enriched GO-terms (*p* < 0.01) with less than 1000 annotated genes among the expressed genes on the microarray. Two leaves were consecutively exposed to feeding *S. exigua* larvae for 24 h and harvested for microarray analysis (*N* = 3). Venn plots for numbers of GO-terms enriched in the (**a**) L0-leaf that had been previously oviposited in E- and EF- but not in F-plants and (**b**) the next younger L1-leaf. The lists of GO-terms enriched in E-plants for each location are included.

We used gene ontology (GO) enrichment analysis to investigate the biological processes in which genes were differentially regulated by the different treatments. In response to feeding (F- and/or EF-plants compared to C-plants), 194 and 293 GO-terms were significantly enriched in the L0- and L1-leaf. F- and EF-plants shared around 60% of these terms (Appendix A: Figure A5), which include many terms related to plant responses to biotic and abiotic stressors, phytohormone biosynthesis and signaling (mostly JA-related), oxidative stress, and plant secondary metabolism (Supplementary material: Table S2). In E-plants only 12 and 19 GO-terms were significantly enriched in the L0- and L1-leaf. These terms did not overlap between both leaf positions and those enriched in the L1-leaf of E-plants were only represented by two–three differentially regulated genes (Supplementary material: Table S2) with only one exception (GO:0018958; phenol-containing compound metabolic process represented by six genes). Half of the 12 GO-terms enriched in the L0-leaf of E-plants (represented by two-nine genes) overlapped with both feeding treatments but two overlapped only with EF-plants (Appendix A: Figure A5).

**Figure 5:**
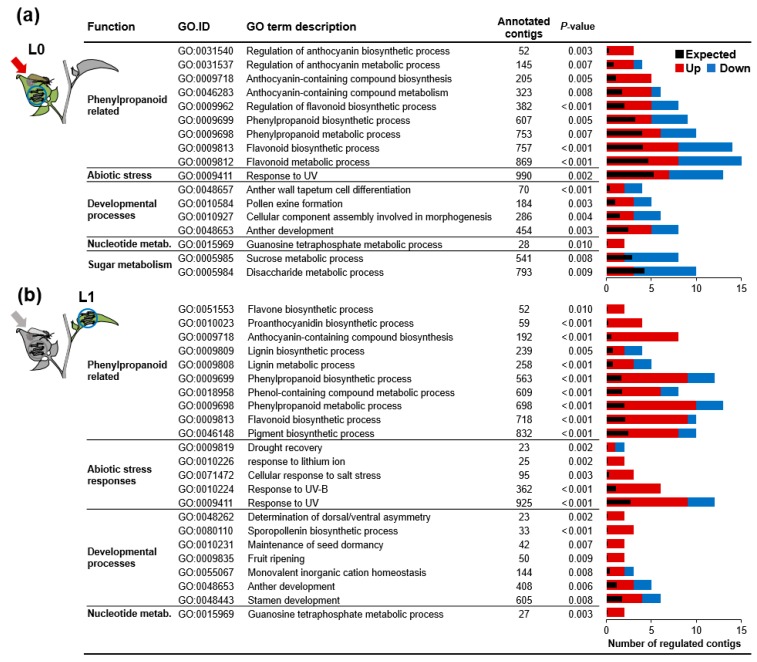
GO-enrichment for genes differing in oviposited and non-oviposited plants after larval feeding. Two leaves of *S. dulcamara* plants were consecutively exposed to feeding *S. exigua* larvae for 24 h and harvested for microarray analysis (*N* = 3). The first leaf position (L0) was previously exposed to *S. exigua* oviposition (Eggs + feeding: EF) or not (Feeding: F). Differentially expressed (log_2_-fold change > 1, *p* < 0.05) genes between F- and EF-plants (**a**) in the L0-leaf or (**b**) the next younger leaf (L1-leaf, which was fed by the larvae one day later) were analyzed for significantly enriched GO-terms (*p* < 0.01). Significant GO-terms with less than 1000 annotated genes among the expressed genes on the microarray are listed and sorted within functional groups (only the smallest term of redundant terms represented by the same gene set are included). For each term, the numbers of up- and down-regulated genes in EF- compared to F-plants are displayed as red and blue bars and black bars show the number of genes expected to be regulated according to the number of genes annotated within each term among the expressed genes.

We then investigated whether genes differentially regulated between F- and EF-plants are overrepresented in distinct biological processes. In the L0-leaf, we found biological processes that can be grouped into five functional categories to be enriched (Figure 5a). Several GO-terms were related to developmental processes that are mainly related to the formation of reproductive tissues. Nevertheless, GO-terms with the highest number of differentially expressed genes were related to synthesis and metabolism of phenylpropanoids such as flavonoids and anthocyanins.

In the GO-analysis of the L1-leaf (systemic to oviposition), phenylpropanoid related processes also showed the largest fraction of differentially expressed genes among the enriched biological process terms (Figure 5b). This functional group was extended by new GO-terms which are processes related to lignin biosynthesis and metabolism. Again, developmental processes were represented as were terms related to abiotic stress. Other than in the L0-leaf, the majority of genes in terms enriched in the L1-leaf were more highly expressed in response to feeding on oviposited plants compared to plants that were subjected to feeding alone.

**Figure 6:**
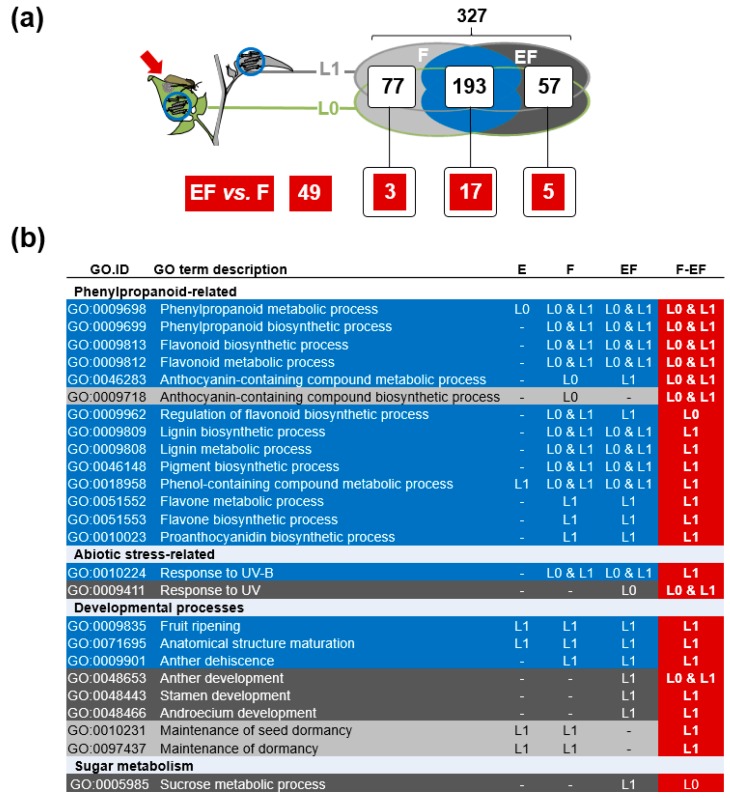
(**a**) Venn plot of GO-terms enriched among genes differentially expressed (log_2_-fold change > 1, *p* < 0.05) relative to control plants and also when directly comparing previously oviposited and non-oviposited *S. dulcamara* plants after feeding by *S. exigua* larvae (Feeding: F; Eggs + feeding: EF). (**b**) List of the significantly enriched GO-terms (*p* < 0.01) with less than 1000 annotated genes among the expressed genes in both types of comparisons. Microarray analyses of two leaves that were consecutively exposed to feeding *S. exigua* larvae for 24 h and harvested for analysis (*N* = 3) are summarized as depicted in (**a**) and the leaf position at which the GO-term was enriched in the different comparisons is given in (**b**).

Finally, we explored which of the biological processes that are regulated after larval feeding in F- and EF-plants are differentially affected in F- and EF-plants. We again summarized the results of the two analyses on the L0- and L1-leaf. Overall 49 different GO-terms were enriched in the comparisons between F- and EF-plants and 25 of these overlapped with terms that were also enriched in either or both (F- and EF-plants) relative to C-plants (Figure 6a). Most of these terms (17) were affected in F- and EF-plants relative to C-plants suggesting that the preceding oviposition altered how these processes are regulated. These terms were again dominated by processes related to phenylpropanoid metabolism while developmental processes that were differentially affected between F- and EF-plants were mostly only altered in either F- or EF-plants (Figure 6b). These analyses further corroborate that insect oviposition may channel how plant phenylpropanoid metabolism is adjusted in response to larval feeding.

### 2.6. Oviposition Alters S. dulcamara’s Metabolic Response to Larval Herbivory

**Figure 7:**
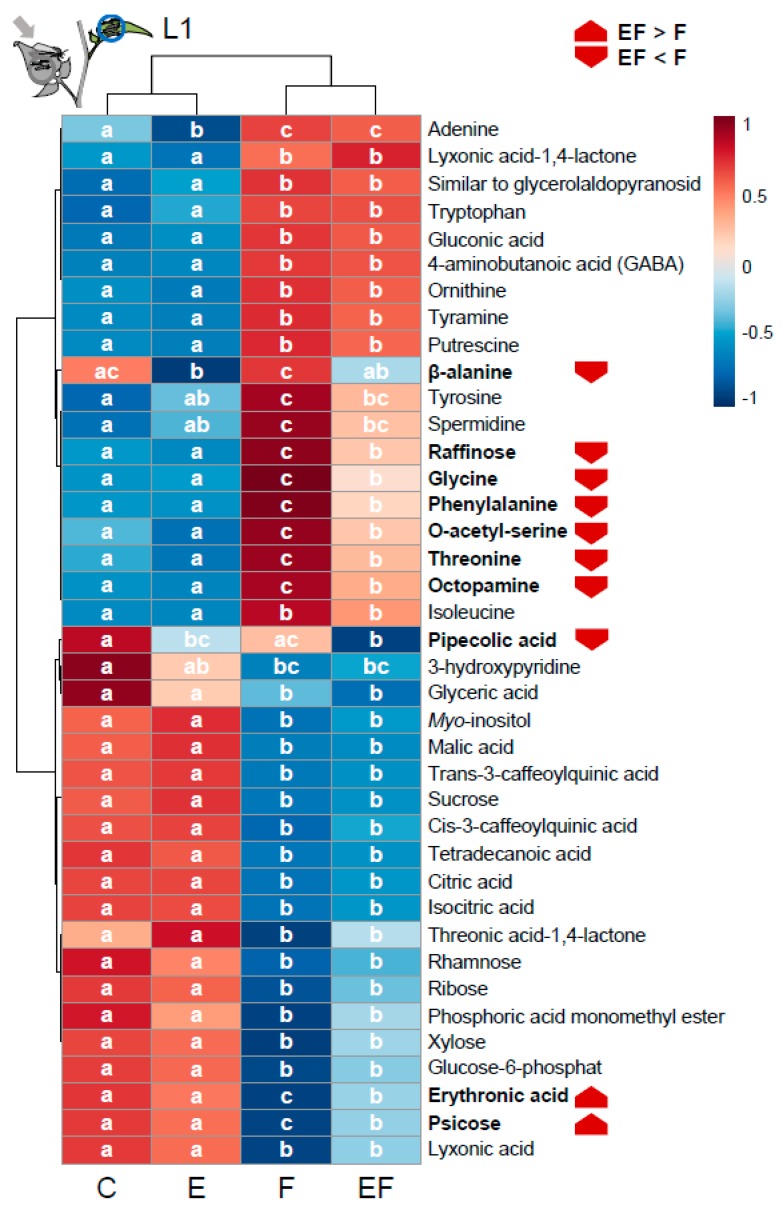
Metabolic response to larval feeding and oviposition. Heat map of leaf metabolite profiles (Pareto-scaled and median-centered normalized peak areas) of control plants (C), plants oviposited by *S. exigua* (E), plants fed by *S. exigua* larvae (F) and oviposited plants fed by larvae (EF). After one day of larval feeding on the previously oviposited leaf or the corresponding leaf of non-oviposited plants, larvae fed for another day on the next-youngest leaf (L1), which was sampled for metabolite profiling. Metabolites that responded to at least one of the treatments according to analysis of variance (ANOVA) following Fisher’s least significant difference (LSD) test for post hoc comparisons are displayed. Different letters in rows indicate significant differences (*p* < 0.05) in metabolite levels between treatments, metabolites of different levels in F- and EF-plants are marked in bold and a red arrow marks the direction of regulation.

In the full-factorial experiment, we further analyzed the L1-leaf in a non-targeted approach for metabolic responses. The levels of 39 metabolites were altered after either previous oviposition, larval feeding or the combination of previous oviposition and feeding (Figure 7). Most of them (90%) were altered in response to larval feeding, while oviposition alone only altered levels of three metabolites (adenine, β-alanine, pipecolic acid). Among the feeding-responsive metabolites are many sugars, amino- and organic acids but also several precursors and intermediates of phenylpropanoid biosynthesis pathways, such as phenylalanine and different caffeoylquinic acids. About half of these metabolites increased after feeding and most of these were amino acids. The feeding induction of four amino acids (glycine, phenylalanine, *O*-acetylserine and threonine), and octopamine, as well as that of raffinose, was significantly lower when oviposition preceded larval feeding. Levels of β-alanine and pipecolic acid that were not feeding-responsive were reduced by oviposition in plants with and without subsequent feeding. *S. dulcamara* showed decreased levels of 20 metabolites in response to feeding larvae, which were mainly organic acids (e.g., malic acid, citric acid, isocitric acid) and sugars (e.g., sucrose, ribose, xylose). The feeding-induced reduction of psicose and erythronic acid was significantly less pronounced when oviposition preceded feeding.

## 3. Discussion

### 3.1. Inferior Performance of S. exigua Larvae on Oviposited S. dulcamara

Our study shows that oviposition by a generalist lepidopteran herbivore on *S. dulcamara* increases the plant’s resistance to subsequently feeding larvae. When feeding on previously oviposited plants, *S. exigua* larvae suffered higher mortality and gained less weight (Figure 1). In general, these results corroborate other studies which found plant-mediated negative effects of insect egg deposition on subsequent tissue-feeding life stages in several plant herbivore systems [11,12,13,14].

Whether oviposition sustainably affected larval weight or mortality depended on the experimental setup. Usually, larvae start feeding gregariously on the leaf on which they hatched and subsequently move to higher leaf positions [30]. When we released larvae to feed freely on the plant after they were confined on standardized leaf positions for two days, their mortality on oviposited plants was increased throughout their entire development. Mortality of larvae confined longer on specific leaves increased quickly, also on non-oviposited plants, but larval weight was lower on oviposited than on non-oviposited plants under such conditions. Larvae released on whole plants gained more weight on oviposited than on non-oviposited plants at day 10. At this time point, larval mortality was almost two-fold increased on oviposited plants and larval weight correlated negatively with the number of surviving larvae (Appendix A: Figure A1). This suggests that the increased mortality on oviposited plants released the larvae from intraspecific competition. Similar to these results, elm leaf beetle larvae feeding upon whole twigs of oviposited elm trees gain more weight than conspecifics on non-oviposited trees and because more larvae die on previously oviposited plants these larvae are also feeding at reduced densities [10]. A previous study showed that growth of *S. exigua* on another *Solanum* species (tomato) is density-dependent and attributed this to the result of intraspecific competition and density-dependent changes in plant quality [31] as, for example, differential activation of PI activity [32].

### 3.2. S. dulcamara’s PI Activity Does Not Explain the Effects of Oviposition on S. exigua Larvae

As oviposition by lepidopteran herbivores primed wound-induced PI activity in *N. attenuata* and PI transcript levels in tomato [11,17], we examined *S. dulcamara*’s inducibility of PI activity (Figure 2). Similar to other solanaceous plants [33,34,35], PI activity in *S. dulcamara* was significantly induced 72 h after onset of feeding by *S. exigua* and fully established after 84 h. However, feeding-induced PI activity was not further increased after *S. exigua* oviposition in *S. dulcamara* after 72 h. Further studies are required to determine defense traits affected by oviposition. For example, *S. dulcamara*’s glycoalkaloid levels, which confer its resistance to slugs [36] could be evaluated.

Future investigations should include standardized induction treatments on oviposited and non-oviposited plants, because effects on herbivore mortality result in divergent feeding intensities. In *N. attenuata*, for example, an increased inducibility of defense traits in oviposited plants is masked due to differential induction intensities when less individuals feed on oviposited plants. Yet, the increased inducibility of phenylpropanoids and PI activity in oviposited plants is apparent when herbivory is mimicked by standardized mechanical wounding and application of the larvae’s oral secretions [11].

### 3.3. No Effects of Oviposition on S. dulcamara’s Phytohormonal Response to One Day of Larval Feeding

In tomato, the oviposition-primed induction of the PI gene was associated with an increased JA burst after wounding of oviposited plants [17]. After 24 h of continuous feeding, we determined no differences in phytohormone levels between oviposited and non-oviposited plants in both analyzed leaf positions (Figure 3). The mechanisms responsible for the increased resistance of oviposited plants may act either independently of the analyzed phytohormones, downstream of their biosynthesis or alter phytohormone induction at other time points. A recent study in elm, for example, found that genes involved in JA metabolism and downstream response genes were more strongly upregulated in previously oviposited plants than in non-oviposited plants as early as 1 h after larval feeding of elm leaf beetle larvae, while this difference between oviposited and non-oviposited plants was not detectable anymore after 24 h [26]. Thus, egg-mediated effects on the plant signaling pathways induced by herbivory can be very fast and future studies have to test at earlier time points whether this is also the case in *S. dulcamara*.

### 3.4. Oviposition Altered S. dulcamara’s Transcriptional Response to Larval Feeding

**Figure A6:**
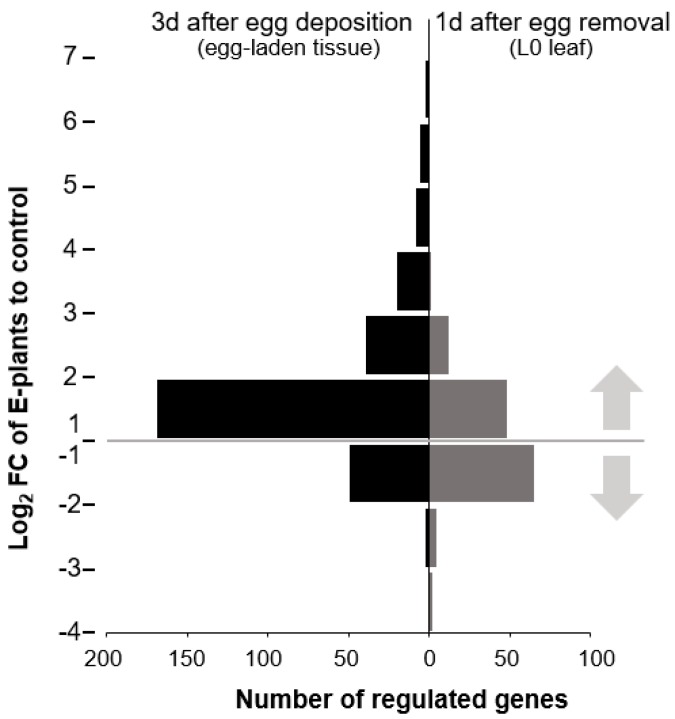
Comparison of regulated genes before and after egg removal. The microarray analyses (*N* = 3) of two experiments with oviposited (Eggs: E) and untreated (Control: C) *S. dulcamara* plants were compared for the numbers of genes regulated to different degrees. Left: Significant expression changes in the RNA extracted from leaf tissue beneath the egg clutches and corresponding tissue of control plants, 72h after oviposition (295 regulated genes according to a previously published analysis, see [6]). Right: Expression changes in the leaf subjected to oviposition (L0) and harvested one day after egg removal in the present study (132 regulated genes). Of the genes regulated in the local tissue one day before the egg incubation time ended, 83% were up-regulated (15% of these to a log_2_FC of more than 3). Only 46% of the genes regulated in the L0-leaf one day after egg-removal were up-regulated and only one of them exceeded to a log_2_FC of 3.

Our non-targeted transcriptome and metabolome analyses revealed that previous oviposition altered *S. dulcamara*’s response to herbivory. This suggests that the plant is not only able to directly respond to the herbivore eggs as shown previously [6], but also to integrate this stimulus when responding to the subsequently feeding life stages. During egg exposure, this earlier study revealed strong transcriptional changes in the oviposited leaf tissue with more than 80% up-regulated genes. One day after egg removal, transcript activation in the previously oviposited L0-leaf was less distinct (Appendix A: Figure A6). Not even half of the genes in the L0-leaf of E-plants were upregulated (not exceeding a log_2_-FC of 3). None of the gene classes with the strongest up-regulation underneath the egg clutch (log_2_-FC of 3 to 7), such as peroxidases, PIs and polyphenol oxidases [6], were differentially expressed between E- and C-plants. In our study, GO-terms related to fatty acid metabolism, in particular, were enriched in the L0-leaf of E-plants (Appendix A: Figure A5). Overall, these results suggest that the response to oviposition had relaxed one day after the eggs had been removed.

Nevertheless, oviposited and non-oviposited plants responded divergently to larval feeding. Relative to control plants, the transcriptional changes in F- and EF-plants overlapped only moderately by about 34% in the previously oviposited and by 50% in a systemic leaf position, while a large fraction was regulated either solely in F- or EF-plants (Figure 4), which is very similar to the 50% overlap between oviposited and non-oviposited *A. thaliana* plants in response to *P. brassicae* [22]. In contrast, the transcriptional response of *B. nigra* to *P. brassicae* herbivory after pre-treatment with egg-extract overlaps to 85% with that of plants without such a pre-treatment [23]. It was proposed that the plant prioritizes the response to the second stress over that to a first stimulus. The much lower overlap between F- and EF-plants suggests that this is not the case for the sequence of oviposition and larval feeding in *S. dulcamara*. As only 7% of the genes solely regulated in EF-plants were also altered in E-plants in the same direction, the divergent responses in F- and EF-plants are not driven by genes regulated directly by the oviposition.

### 3.5. Oviposition Altered the Transcriptional Regulation of Phenylpropanoids in Response to Larval Feeding

When we directly compared the transcriptomes of F- and EF-plants, we could demonstrate differential expression for 240 genes, which is in the same range as the 220 genes found in a similar analysis in *A. thaliana* [22]. The proportion of genes significantly different between F- and EF-plants was highest among genes solely down-regulated in EF-plants (23%), while in absolute numbers most differentially regulated genes were found among the genes solely down-regulated in F-plants. Although twice as many genes differed between F- and EF-plants in the L0-leaf than in the L1-leaf, we found in both leaf positions very similar GO terms enriched within the differentially regulated genes (Figure 5). In the L1-leaf, these GO-terms were more strongly dominated by up-regulated genes than in the previously oviposited L0-leaf suggesting an activation of these biological processes upon feeding that occurs also in leaves systemic to oviposition.

Besides developmental processes mainly related to reproductive organ formation, in both leaf positions processes related to phenylpropanoid biosynthesis and metabolism represented a prominent group of the GO-terms enriched in EF compared to F-plants. The fact that most of these terms were enriched in both F- and EF-plants (Figure 6) but were still enriched in a direct comparison between the two treatments suggests that oviposition may shift feeding-induced phenylpropanoid biosynthesis and metabolism. These processes were dominated by biosynthesis/metabolism of anthocyanins, flavones and lignin. Several compounds of these chemical classes are known to improve plant resistance to herbivores [37,38]. Our results in *S. dulcamara* nicely parallel other recent studies in different plant species that found the feeding-induced levels of phenylpropanoids to be altered through a previous insect oviposition. The priming effect of *S. exigua* oviposition on *N. attenuata* results in an increased feeding-induced accumulation of the phenylpropanoid caffeoylputrescine in leaves systemic to the oviposited leaf, and biosynthesis of such phenylpropanoids is required for oviposition-mediated plant resistance against the subsequent larval attack [11,39]. Moreover, in elm and *A. thaliana*, phenylpropanoids of the flavonoid class have been proposed to be involved in oviposition-primed resistance against elm leaf beetle and *P. brassicae* larvae, respectively [10,22].

### 3.6. Oviposition-Mediated Modifications of Feeding-Induced Changes of Amino Acid and Sugar Metabolism

Our metabolome analysis revealed a range of primary metabolites that were altered in *S. dulcamara* in response to feeding *S. exigua* larvae (Figure 7). Herbivory-induced changes of plant primary metabolites are generally assumed to result from (i) altered resource allocation, (ii) shifts into secondary metabolite production, (iii) their involvement in defense signaling, or (iv) acting as defense themselves [40]. About half of the herbivory-responsive metabolites, mostly amino acids, were increased after feeding. These included 4-aminobutanoic acid (GABA), several amines (tyramine, putrescine and octopamine), 3-phosphglycerate-derived amino acids (glycine and O-acetyl-serine) and shikimate-derived amino acids (tyrosine, phenylalanine, and tryptophan). These are precursors for a large variety of defensive secondary metabolites, such as different phenolics and alkaloids, but also for phytohormones such as auxin and JA [41]. For example, phenylalanine is the starting point of phenylpropanoid biosynthesis [42], glycine is suggested as a precursor for glycoalkaloid biosynthesis [43,44] and tryptophan is a precursor for auxin and indole, which can prime anti-herbivore defense in maize [45]. Thus, upon herbivore attack, plants may activate an amino acid metabolism to supply the precursors of the pathways producing defensive secondary metabolites. When oviposition preceded larval feeding, the induction of several amino acids (phenylalanine, glycine, O-acetyl-serine, threonine) and octopamine was attenuated. Since in parallel, oviposition especially increased the feeding-induction of genes related to phenylpropanoid metabolism, a larger exploitation of the precursors of defensive metabolites is likely. The observed metabolic changes may hint towards an important role of primary metabolism for priming of stress responses as indicated recently [45].

Metabolites with reduced levels in feeding-induced plants were dominated by sugars and tricarboxylic acid (TCA)-cycle intermediates (e.g., malic acid, citric and isocitric acid). This could mark a deficiency in assimilates due to reduced photosynthesis in response to larval feeding, which is apparent in many plant species including *S. dulcamara* [46] and due to carbon reallocation from the attacked to non-attacked tissues as evidenced in some plant species [47,48]. Additionally, if increased amino acid metabolism fed into secondary metabolite production as discussed above, it could deter glycolysis intermediates from entering the TCA-cycle. GABA levels increased along with diminished intermediates of glycolysis and the TCA-cycle. This may signify that the plant uses succinate from the GABA metabolism as an alternative source to maintain the TCA-cycle, which is known as the GABA shunt [49]. Apart from glutamate, GABA can also be synthesized from putrescine and spermidine, which both increase after feeding. GABA can also act as a direct, JA-independent anti-herbivore defense as reported for *A. thaliana* [50].

Among the metabolites reduced in feeding-induced plants were also intermediates of the core phenylpropanoid metabolism, i.e., *cis*- and *trans*-3-caffeoyl-quinic acid, known as chlorogenic acid. Chlorogenic acid is a defense compound and gives rise to various metabolic derivatives that also act in defense. Again, this could be a consequence of an increased turnover to produce these compounds that are widely accepted as anti-herbivore defenses [51,52,53]. Although several of the metabolites reduced after feeding tended to be less reduced when oviposition preceded, this was only significant for two metabolites. Additionally, two compounds were not altered by feeding but reduced in response to oviposition. Besides β-alanine, this was the case for pipecolic acid, a major regulator of systemic acquired resistance (SAR) against phytopathogens [54]. In *A. thaliana*, egg-extract treatments actually induced SAR and also levels of pipecolic acid [55]. In contrast to *A. thaliana*, in *S. dulcamara* we found no signs of SAR activation by insect oviposition in the transcriptomic analyses before [6] and after the natural egg incubation time.

### 3.7. Conclusion

It is well established that plants perceive and respond to oviposition by herbivorous insects and that this can affect the interaction of the plant with the herbivorous life stage, the larvae [5]. We demonstrated that insect oviposition on *S. dulcamara* increases the plant’s resistance to the feeding larvae just as had been previously observed in other plant species [11,12,13,14]. Yet, how plant responses to larval herbivory are affected by prior oviposition is largely unknown. We show that *S. exigua* oviposition on *S. dulcamara* alters its transcriptional response to larval feeding in leaves local and systemic to oviposition, which was reflected by reciprocal changes at the level of primary metabolites. Although further studies will be required to identify signaling events and defense traits that mediate the plant resistance to the larvae, we provide here evidence that phenylpropanoids are promising candidates for oviposition-primed defense responses in *S. dulcamara*.

## 4. Materials and Methods

### 4.1. Plants and Insects

We used *Solanum dulcamara* L. (Solanaceae) plants originating from different populations in the vicinity of Berlin (Erkner: 52° 25′ 07.3″ N; 13° 46′ 26.2″ E, Grunewald: 52° 27′ 44.37″ N; 13° 11′ 24.63″ E, and Siethen 52° 16′ 53.65″ N; 13° 11′ 18.65″ E) and from the Netherlands (Friesland: 52° 58′ 36.2” N 5° 30′ 59.4” E and Gooree 51° 49′ 23.8” N; 3° 53′ 19.2″ E; accession numbers B24750030 and B24750010 of the Solanaceae Genebank at Radboud University (http://www.ru.nl/bgard/). Plants were grown in 0.75 L pots with a standard soil (Einheitserde^®^, type: Profi Substrate Classic, Sinntal-Jossa, Germany) in a greenhouse (20–25 °C, 16/8 h light/dark) with ample water supply. We used 3–4-week-old plants grown from stem cuttings of 6–7-week-old plants as described previously [6], except in one experiment for which plants were grown from seeds (see below).

In all experiments, we used similar-sized plants of the same genotype for each replicate block consisting of all treatments. Experimental plants received either *S. exigua* eggs by natural oviposition (Eggs: E), received no eggs but feeding (Feeding: F), received both, egg deposition and feeding (Eggs + feeding: EF), or remained untreated (Control: C).

*Spodoptera exigua* HÜBNER (Noctuidae) larvae were reared in vented plastic boxes (14 × 21 × 5 cm) on a bean flour-based artificial diet as described previously [46]. Boxes were kept in a climate chamber (24 °C, 70% relative humidity, 16/8 h light/dark with 50% dimming for 1 h). The moths were kept in flight cages supplied with 20% honey solution and paper tissue as substrate for oviposition.

### 4.2. Oviposition and Herbivory Treatments

We exposed a defined leaf position (the 5^th^ fully developed leaf) to *S. exigua* oviposition through slits in a flight cage (40 × 71 × 40 cm) with 40 female and 40 male moths overnight. Control plants were exposed to 80 male moths. We obtained oviposited plants with an egg load of 10–150 eggs. Shortly before the larvae would hatch after 3–4 days the eggs turn black and were carefully removed using a moistened paint brush to avoid uncontrolled larval feeding. Then, a defined number of neonate larvae (see below) was transferred to the leaf previously carrying the eggs and enclosed in vented clip cages. Control plants received empty clip cages.

### 4.3. Larval Performance and Feeding Damage

In two experiments with only 2 treatments (F and EF), 6 neonate larvae per plant were allowed to feed for 6 days upon egg removal. Every other day, we transferred larvae to the next younger leaf and recorded larval mortality. At day 4 and 6, we additionally determined mean weight of all larvae per plant.

In another experiment with 6-week-old plants grown from seeds, 12 neonate larvae per plant started feeding 12 h after egg removal. This full-factorial experiment included all 4 treatments (C, E, F, EF). Encaged in clip cages, the larvae fed on the previously egg-laden leaf on EF-plants or the corresponding leaf position of F-plants for 24 h. Then, this leaf and the corresponding leaf of unfed C- and E-plants was cut at the petiole and immediately flash frozen in liquid nitrogen for phytohormone, transcriptome and metabolome analyses (L0-leaf). The larvae were transferred to the adjacent upper leaf, which was similarly harvested another 24 h later (L1-leaf). Subsequently, the larvae were allowed to feed freely on the whole plant that was enclosed in a gauze bag for another 8 days. Surviving larvae from each plant were weighted, transferred to plastic boxes with artificial diet and kept in a climate chamber (24 °C, 70% relative humidity, 16/8 h light/dark with 50% dimming for 1 h) until pupation to record mortality and mean pupal weight 1 day after pupation. Each time the larvae were transferred, we recorded larval survival. At day 10, we took photographs to determine the total and damaged leaf areas. The excised leaves were pictured on a white panel with four reference areas of 1 cm^2^ in each corner. We used Photoshop CS5 (Adobe Systems, San Jose, CA, USA) to receive binary images of the consumed leaf area which was quantified with “ImageJ 1.47v” relative to the reference areas (U.S. National Institutes of Health, Bethesda, MD, USA, https://imagej.net).

### 4.4. Induction of PI Activity by Larval Feeding

To first establish the inducibility of trypsin PI activity in *S. dulcamara* by larval feeding and JA, we applied to the fourth fully developed leaf either a third-instar *S. exigua* larva (pre-reared on *S. dulcamara* leaves) or 150 µg of the methyl ester of jasmonic acid (MeJA; Sigma-Aldrich, Darmstadt, Germany) or left it untreated. The MeJA was applied in 20 µL lanolin spread with a spatula on the basal quarter of the leaf lamina and control plants received a similar treatment without MeJA. After two days, the larvae were removed and the treated leaves (the parts not covered by lanolin) of the first set of plants of all treatments were harvested and flash frozen in liquid nitrogen. Three other sets were harvested another 24 h, 36 h (which was in the dark phase) and 48 h later.

We then exposed in a full-factorial experiment previously egg-laden leaves of EF-plants and corresponding leaf positions F-plants to 20 neonate *S. exigua* larvae a day after egg removal. The encaged larvae were allowed to feed for 72 h on F- and EF-plants before this leaf and the corresponding leaf of unfed C- and E-plants was harvested into liquid nitrogen.

### 4.5. Extraction and Quantification of PI Activity.

Extraction of the leaf samples and radial diffusion assays to measure trypsin PI activity were performed as described previously [35] with minor modifications. The powdered leaf material was weighed and approximately 100 mg was extracted with 300 µL extraction buffer and the supernatant after centrifugation was applied to agar plates with trypsin (from bovine pancreas; Sigma-Aldrich, Darmstadt, Germany). On each plate a dilution series of a soybean trypsin protease inhibitor (Sigma- Aldrich, Darmstadt, Germany) was run as standard curve. After 15–17 h of diffusion time at 4 °C, plates were incubated at 37 °C with *N*-acetyl-dl-phenyl-alanine β-naphtyl ester (Sigma- Aldrich, Darmstadt, Germany) that served as substrate for trypsin activity and the diazo stain fast blue (Sigma-Aldrich, Darmstadt, Germany). Stained plates were photographed. We determined the Feret’s diameters of the inhibition zones with UTHSCSA ImageTool^®^ (University of Texas Health Science Center, San Antonio, TX, USA) in binary images retrieved through Photoshop CS5. Trypsin PI activity was then calculated according to the corresponding standard curves and divided by the total protein content as determined in a Bradford assay run in micro titer plates with Roti^®^quant (Carl Roth, Karlsruhe, Germany) and albumin as protein standard according to the manufacturer’s instructions.

### 4.6. RNA Extraction and Microarray Analysis

Total RNA was extracted from 100 mg of powdered leaf tissue (L0- and L1-leaves) with the NucleoSpin^®^ RNA Plant kit (Macherey-Nagel GmbH & Co. KG, Düren, Germany) following the manufacturer’s instructions using double the amount of RAP lysis buffer. Genomic DNA was removed using TURBO DNA-free™ (Thermo Fisher Scientific, Waltham, MA, USA) according to the manufacturer’s instructions. RNA samples of 3 plants were pooled to obtain 3 biological replicates before hybridization on an 8 × 60K Agilent custom microarray for *S. dulcamara* (NCBI GEO platform GPL23228) described earlier [46].

RNA labelling, hybridization and scanning of the arrays was performed by Oaklabs (Henningsdorf, Germany). Array data processing and analysis were performed with the “limma” software package from Bioconductor in “R” (R Core Team; [56]) as described earlier [46]. Probes not exceeding the fluorescence values of the 90% percentile of dark-corners by 1.5- or 1.8-fold (in the analysis of the L0- or L1-leaves respectively) were considered as non-expressed and excluded from further analysis. Then, the data were background-corrected and normalized using the “normexp” and “quantile” methods. Multiple oligos matching the same target sequence at the same strand were averaged.

Gene ontology enrichment analysis in biological processes was performed using an annotation described previously [28] and the package “topGo” [57]. The GO distribution in the set of targets that were differentially expressed between *S. exigua* larval feeding with and without previous oviposition was compared to the GO distribution of all targets included in the data analysis using the “elim” algorithm at a minimum node size of 20. Fisher’s exact tests were used to generate *p*-values for the enrichment of each GO term.

### 4.7. Reverse Transcription and qPCR Analysis

Four of the genes significantly different between F and EF plants on the microarray were analyzed in the RNA of all individual plants by qPCR analysis as described previously [11]. In brief, cDNA was synthesized with the Reverse Transcriptase Core kit and subjected to SYBR^®^Green-based real-time PCR using the qPCR core kit (bothta kits: Eurogentec, Seraing, Belgium, http://www.eurogentec.com) and gene specific primers (Supplementary Material: Table S3) on a Stratagene™ Mx3005P^®^ instrument (Agilent Technologies, Santa Clara, CA, USA, http://www.agilent.com).

### 4.8. Phytohormone Extraction and Quantification

Leaf tissue samples were analyzed for JA, JA-Ile, SA and ABA content using a LC-MS/MS based method as described previously [28]. About 100 mg powdered leaf material was extracted twice in 1ml ethyl acetate using a FastPrep homogenizer (MP Biomedicals, Solon, OH, USA) and 1.25 g homogenization matrix (Zirconox, 2.8–3.3 mm; Mühlmeier Mahltechnik, Bärnau, Germany). The ethyl acetate of the first extraction was spiked with internal standards (20ng of D4-SA, 60.4 ng D6-JA, 20 ng D6-JA-Ile, 20 ng D6-ABA). The combined supernatants were dried in a vacuum concentrator (concentrator 5301, Eppendorf, Hamburg, Germany) and the residue was re-eluted in 400 µL 70% methanol containing 0.1% formic acid (*v*/*v*) by vortexing. For analysis of phytohormones by UPLC-ESI-MS/MS (Synapt G2-S HDMS; Waters^®^, Milford, MA, USA), 7 µL of the supernatant was separated on a C 18 column (Acquinity UPLC BEH-C18, ø 2.1 × 50 mm, particle size 1.7 µm) with solvent A water and solvent B methanol (both containing 0.1% formic acid) in a gradient mode (eluent B: 0 min: 30%; 1 min: 30%; 4.5 min: 90%; 8 min: 90%; 9 min: 30%; 3 min equilibration time between runs) at a flow rate of 250 µL/min. Compounds were detected in negative ionization mode with parent ion/daughter ion selections of 209/59 for JA, 322/130 for JA-Ile 137/93 for SA, 263/153 for ABA, 215/59 for D6-JA, 328/130 for D6-JA-Ile, 141/93 for D4-SA and 269/159 for D6-ABA. Phytohormones were quantified using MassLynx™ Software (version 4.1; Waters, Milford, MA, USA) according to peak areas of the respective fragment ions relative to the internal standards.

### 4.9. Non-Targeted Metabolome Analysis

Profiling of primary metabolites was performed as previously described [58]. Frozen leaf tissue (~100 mg) was ground (2 × 45 s, maximum frequency) by a Retsch mill (MM 400, Retsch, Haan, Germany). Per mg of leaf tissue, metabolites were extracted with 4.5 µL methanol containing 0.2 mg · mL^-1^ U-13C-sorbitol as internal standard at 70 °C for 15 min, then 2.5 µL chloroform was added for 5 min at 37 °C. The liquid was partitioned by adding 5 µL H_2_O per mg leaf tissue to obtain polar metabolites. The dried polar fraction (~160 µL) was derivatised by methoxyamination and trimethylsilylation. A mixture of n-alkanes (C12, C15, C18, C19, C22, C28, C32 and C36) served as retention index standards [59]. A 1 µL aliquot of the samples was injected in splitless mode at 230 °C into a 6890N24 gas chromatograph (Agilent Technologies, Böblingen, Germany; http://www.agilent.com). The sample was separated on a Varian Factor Four column (VF-5ms, length 30 m, diameter 0.25 mm, and 0.25 µm film thickness) (Agilent Technologies, Böblingen, Germany) using the following temperature program: 1 min at 70 °C; ramp to 350 °C at 9°/min, 5 min at 350 °C, then cooling. Compounds were detected by electron ionization/time-of-flight mass spectrometry (EI-TOF-MS) using a Pegasus III TOF mass spectrometer (LECO Instrumente GmbH, Mönchengladbach, Germany). Chromatograms were obtained and baseline corrected by ChromaTOF software (Version 4.22, LECO, St. Joseph, MO, USA). Identification of metabolites was manually supervised with the TagFinder software [60] and the mass spectra and retention time index (RI) reference collection of the Golm Metabolome Database [61,62]. Peak heights were normalized to fresh weights.

### 4.10. Statistical Analysis

All statistics were performed with R software, version 3.2.3 [63] and MetaboAnalyst 3.0 [64]. Ratio scaled data were graphically checked for normal distribution using Q-Q plots.

The data of both experiments assessing performance of 6 neonate larvae per plant were combined for analysis and all models were first run with the experimental repetition as factor but since it had no significant effect it was not included in the final models. We tested for an effect of previous oviposition on larval mortality with generalized linear mixed models (GLMMs) (function “lmer” in package “lme4”, [65]) with binomial error distribution and a logit link function, and included plant population identity as random factor. Larval and pupal masses on egg experienced und unexperienced plants were compared with linear mixed models (LMMs) including replicate block as random factor.

We compared PI activity in leaves of plants fed by a single *S. exigua* larva and MeJA-induced plants by Welch’s *t*-tests to the contemporary harvested control plants at each time point.

We analyzed data sets for PI activity and phytohormones from the two-factorial experiments with LMMs with oviposition and feeding as fixed factors and replicate block as random factor. All summaries of the statistical models described above are provided in Supplementary Table S4–S9.

For microarray analysis we log_2_-transformed the average fluorescence values of the probes for each gene and fit them to a linear model using the “lmFit” function. Targets differing at least 2-fold between treatments with a *p*-value below 0.05 were considered significantly different. To explore the overlaps in genes regulated by feeding alone (F- versus C-plants), feeding on oviposited plants (EF- versus C-plants), oviposition alone (E- versus C-plants) and the genes with a significantly different expression in fed plants with and without prior oviposition (F- versus EF-plants), we used Venny [66].

We used pairwise comparisons for the log_2_-transformed expression values to evaluate the qPCR analysis data.

The metabolome data were analyzed using MetaboAnalyst 3.0. The data were cube root-transformed, Pareto scaled and median centered, and treatment effects on metabolite levels were estimated with ANOVA followed by Fisher’s LSD test for post hoc comparisons.

## Supplementary Materials

Supplementary materials can be found at http://www.mdpi.com/1422-0067/19/12/4008/s1. Microarray raw data are available at NCBI Gene Expression Omnibus (GEO accession: GSE123538).

## Abbreviations

ABA	Abscisic acid
GABA	4-amino butanoic acid
FC	Fold-change
GC-MS	Gas chromatography coupled to mass spectrometry
GLMM	generalized linear mixed model
GO	Gene ontology
GST	Glutathione S-transferase
HQT	Hydroxycinnamoyl-CoA quinate transferase
JA	Jasmonic acid
JA-Ile	Jasmonic acid isoleucine
MDPI	Multidisciplinary Digital Publishing Institute
MeJA	Methyl jasmonate
LMM	Linear mixed models
LSD	Least significant difference
PI	Protease inhibitor
SA	Salicylic acid
SE	Standard error
TOF-MS	Time-of-flight mass spectrometry
