# Supplementary material for: Oviposition by Spodoptera exigua on Solanum dulcamara Alters the Plant’s Response to Herbivory and Impairs Larval Performance
Source: Int J Mol Sci. 2018 Dec 12;19(12):4008. doi: 10.3390/ijms19124008 (PMC6321313; doi:10.3390/ijms19124008)
Supplement: Supplementary file 1 [file ijms-19-04008-s001.zip › Supplementary Materials.pdf]

Article title: **Oviposition by *Spodoptera exigua* on *Solanum dulcamara* alters the plant's response to herbivory and impairs larval performance**

Authors: Daniel Geuss, Tobias Lortzing, Jens Schwachtje, Jens Schwachtje and Anke Steppuhn

Supplementary Materials

Table S1. Complete lists of all differentially regulated genes between any of the treatments in either of the two leaves analyzed by microarrays are provided in the Exel file " Supplementary Table S1".

Table S2. Complete lists of GO-term lists enriched among the differentially regulated genes between any of the treatments in either of the two leaves analyzed by microarrays are provided in the Exel file "Supplementary Table S2".

Table S3. Primers used for qPCR of *S. dulcamara* genes.

Gene name	Accession		Sequence (5'-3')
GST	comp17002_c0_seq1	F	ACGAACAAAGGCTGTCCAAG
		R	CCTAAGGCTAGGGAGATGGC
HQT	comp16609_c0_seq1	F	AGAAGCCTAATGCGTGTGGT
		R	TCTGGATCTTGGTTGCTCTG
ANS	comp14387_c0_seq1	F	CTTCTGGCAATGTCCAAGGC
		R	CCTCGGGGAAAACACAATGG
MLP	comp17996_c0_seq1	F	TGTGAACACGAGTGGACTACAT
		R	AGCAAAACACCAAAGGGCAA

Table S4. Statistical models assessing the effect of oviposition on performance parameters of *S. exigua* larvae kept on defined *S. dulcamara* leaves. Summaries of generalized linear mixed models (GLMMs) testing the effect of oviposition on larval mortality after (**a**) two days, (**b**) four days, (**c**) six days of feeding and of linear mixed models (LMMs) testing the effect of oviposition on larval mass after (**d**) four days and (**e**) six days. Significant P-values are highlighted in bold.

**(a) GLMM fit by ML for Binomial- distributed data of dead to live larvae day 2**				
Random effects	**variance**	***SD***		
Population	0.10	0.31		
Fixed effects	**estimate**	***SE***	***Z***	***P***
Intercept	-2.65	0.36	-7.44	**< 0.001**
Eggs (yes/no)	1.47	0.38	3.84	**< 0.001**

**(b) GLMM fit by ML for Binomial- distributed data of dead to live larvae day 4**				
Random effects	**variance**	***SD***		
Population	0.08	0.28		
Fixed effects	**estimate**	***SE***	***Z***	***P***
Intercept	-1.16	0.23	-5.08	**< 0.001**
Eggs (yes/no)	0.59	0.26	2.274	**0.023**

**(c) GLMM fit by ML for Binomial- distributed data of dead to live larvae day 6**				
Random effects	**variance**	***SD***		
Population	0.48	0.69		
Fixed effects	**estimate**	***SE***	***Z***	***P***
Intercept	-0.22	0.34	-0.67	0.506
Eggs (yes/no)	0.24	0.24	0.97	0.332

**(d) LMM fit by REML for larval mass on day 4**				
Random effects	**variance**	***SD***		
Replicate	0.29	0.54		
Fixed effects	**estimate**	***SE***	***Z***	***P***
Intercept	0.99	0.12	8.14	**< 0.001**
Eggs (yes/no)	-0.15	0.08	-1.92	0.055

**(e) LMM fit by REML for larval mass on day 6**				
Random effects	**variance**	***SD***		
Replicate	1.42	1.19		
Fixed effects	**estimate**	***SE***	***Z***	***P***
Intercept	3.86	0.38	10.12	**< 0.001**
Eggs (yes/no)	-0.96	0.43	-2.23	**0.026**

Table S5. Statistical models assessing the effect of oviposition on performance parameters of *S. exigua* larvae released on *S. dulcamara* plants. Summaries of generalized linear mixed models (GLMMs) testing the effect of oviposition on larval mortality after (**a**) one day, (**b**) two days, (**c**) ten days after release on the plant and (**d**) until pupation and of linear mixed models (LMMs) testing the effect of oviposition on mass of larvae after ten days on the plant and of pupae developing from these larvae. Significant P-values are highlighted in bold.

**(a) GLMM fit by ML for Binomial- distributed data of dead to live larvae day 1**				
Random effects	**variance**	*SD*		
Replicate	0.37	0.61		
Fixed effects	**estimate**	*SE*	*Z*	*P*
Intercept	-2.13	0.39	5.45	**< 0.001**
Eggs (yes/no)	-0.42	0.46	-0.91	0.36
**(b) GLMM fit by ML for Binomial- distributed data of dead to live larvae day 2**				
Random effects	**variance**	*SD*		
Replicate	0.26	0.51		
Fixed effects	**estimate**	*SE*	*Z*	*P*
Intercept	-1.50	0.01	-205.10	**< 0.001**
Eggs (yes/no)	-0.78	0.01	-106.9	**< 0.001**
**(c) GLMM fit by ML for Binomial- distributed data of dead to live larvae day 10**				
Random effects	**variance**	*SD*		
Population	0.18	0.42		
Fixed effects	**estimate**	*SE*	*Z*	*P*
Intercept	0.43	0.25	-1.76	0.079
Eggs (yes/no)	-0.82	0.31	-2.66	**0.008**
**(d) GLMM fit by ML for Binomial- distributed data of dead to live larvae day until**				
Random effects	**variance**	*SD*		
Population	0.05	0.23		
Fixed effects	**estimate**	*SE*	*Z*	*P*
Intercept	-0.04	0.21	-0.18	0.857
Eggs (yes/no)	-0.71	0.28	-2.49	**0.013**
**(e) LMM fit by REML for larval mass on day 10**				
Random effects	**variance**	*SD*		
Replicate	107.20	10.36		
Residuals	178.60	13.37		
Fixed effects	**estimate**	*SE*	*Z*	*P*
Intercept	70.45	5.64	12.50	**< 0.001**
Eggs (yes/no)	-17.10	6.30	-2.71	**0.007**
**(f) LMM fit by REML for pupal mass**				
Random effects	**variance**	*SD*		
Replicate	29.27	5.41		
Residuals	72.84	8.54		
Fixed effects	**estimate**	*SE*	*Z*	*P*
Intercept	118.82	3.37	35.28	**< 0.001**
Eggs (yes/no)	-4.24	4.02	-1.05	0.059

Table S6. Statistical comparisons to assess the inducibility of protease inhibitor (PI) activity in *S. dulcamara* leaves. Welch's t-tests on PI activity data (**a**) 0, (**b**) 24, (**c**) 36 and (**b**) 48 hours after two days of *S. exigua* feeding or treatment with methyl jasmonate (MeJA) at the time the larvae started to feed in comparison to untreated control plants. Significant P-values are highlighted in bold.

**a) Welch's *t*-tests on PI data 0 hours after treatment**			
Comparison	***t***	**df**	***P***
Control-*S. exigua*	-1.34	11.13	0.207
Control-MeJA	-1.81	10.31	0.100
**b) Welch's *t*-tests on PI data 24 hrs after treatment**			
Comparison	***t***	**df**	***P***
Control-*S. exigua*	-2.78	8.00	**0.024**
Control-MeJA	-6.9449	6.00	**< 0.001**
**c) Welch's *t*-tests on PI data 36 hrs after treatment**			
Comparison	***t***	**df**	***P***
Control-*S. exigua*	-5.283	8.00	**< 0.001**
Control-MeJA	-7.278	6.00	**< 0.001**
**d) Welch's *t*-tests on PI data 48 hrs after treatment**			
Comparison	***t***	**df**	***P***
Control-*S. exigua*	-3.696	8.00	**0.006**
Control-MeJA	-5.636	6.00	**0.001**

Table S7. Linear model assessing the effect of *S. exigua* oviposition and feeding on PI activity in *S. dulcamara*. Summary of the linear model testing the effect of *S. exigua* oviposition and feeding on PI activity in *S. dulcamara* leaf tissue. Significant P-values are highlighted in bold.

**LM fit by ML on PI data**				
Fixed effects	**estimate**	*SE*	*t*	***P***
Intercept	0.02	0.04	0.49	0.630
Eggs (yes/no)	0.03	0.05	0.54	0.596
Feeding(yes/no)	0.20	0.05	4.26	**< 0.001**
Eggs x Feeding	0.01	0.07	0.11	0.912

Table S8. Linear mixed models assessing the effect of *S. exigua* oviposition and feeding on phytohormone levels in *S. dulcamara*. Summary of the linear mixed models testing the effect of *S. exigua* oviposition and feeding on levels of: (**a**-**b**) JA; (**c**-**d**) JA-Ile; (**e**-**f**) SA and (**g**-**h**) ABA in *S. dulcamara* leaf tissue. Tissue of the previously oviposited leaf or the same leaf position of non-oviposited plants (L0-leaf) as well as the next younger leaf (L1-leaf) was analyzed. Significant P-values are highlighted in bold.

**(a) LMM fit by ML on JA data of the L0-leaf local to oviposition**				
Random effects	**variance**	*SD*		
Replicate	230.40	15.18		
Fixed effects	**estimate**	*SE*	*t*	*P*
Intercept	0.67	11.63	0.06	0.950
Eggs (yes/no)	-1.60	14.82	-0.11	0.914
Feeding(yes/no)	55.36	14.34	3.86	**< 0.001**
Eggs x Feeding	-2.93	20.29	-0.15	0.885
**(b) LMM fit by ML on JA data of the L1-leaf systemic to oviposition**				
Random effects	**variance**	*SD*		
Replicate	0.00	0.00		
Fixed effects	**estimate**	*SE*	*t*	*P*
Intercept	2.46	22.52	0.11	0.913
Eggs (yes/no)	-0.66	34.04	-0.02	0.985
Feeding(yes/no)	166.50	31.85	5.23	**< 0.001**
Eggs x Feeding	-11.53	46.62	-0.25	0.805
**(c) LMM fit by ML on JA-Ile data of the L0-leaf local to oviposition**				
Random effects	**variance**	*SD*		
Replicate	1.28	1.13		
Fixed effects	**estimate**	*SE*	*t*	*P*
Intercept	0.37	0.69	0.54	0.586
Eggs (yes/no)	0.15	0.81	0.19	0.853
Feeding(yes/no)	5.17	0.79	6.58	**< 0.001**
Eggs x Feeding	-0.73	1.11	-0.66	0.509
**(d) LMM fit by ML on JA-Ile data of the L1-leaf systemic to oviposition**				
Random effects	**variance**	*SD*		
Replicate	1.49	1.22		
Fixed effects	**estimate**	*SE*	*t*	*P*
Intercept	0.57	1.75	0.32	0.746
Eggs (yes/no)	-0.06	2.58	-0.02	0.983
Feeding(yes/no)	10.66	2.41	4.43	**< 0.001**
Eggs x Feeding	-2.84	3.53	-0.81	0.421
**(e) LMM fit by ML on SA data of the L0-leaf local to oviposition**				
Random effects	**variance**	*SD*		
Population	1196.00	34.59		
Fixed effects	**estimate**	*SE*	*t*	*P*
Intercept	29.98	36.58	0.82	0.412
Eggs (yes/no)	47.67	43.39	1.10	0.272
Feeding(yes/no)	29.81	42.07	0.71	0.479
Eggs x Feeding	-10.03	59.53	-0.17	0.866

(Continues)

(f) LMM fit by ML on SA data of the L1-leaf systemic to oviposition

Random effects	**variance**	***SD***		
Population	13133.00	114.60		
Eggs (yes/no)	24.06	71.92	0.33	0.738
Feeding(yes/no)	-26.41	67.14	-0.39	0.694
Eggs x Feeding	44.00	98.93	0.45	0.655

(g) LMM fit by ML on ABA data of the L0-leaf local to oviposition

Random effects	**variance**	***SD***		
Replicate	27.41	5.24		
Fixed effects	**estimate**	***SE***	***t***	***P***
Intercept	53.34	6.73	7.93	**< 0.001**
Eggs (yes/no)	-4.13	9.19	-0.45	0.654
Feeding(yes/no)	18.47	8.91	2.07	**0.038**
Eggs x Feeding	2.50	12.61	0.20	0.843

(h) LMM fit by ML on ABA data of the L1-leaf systemic to oviposition

Random effects	**variance**	***SD***		
Replicate	177.00	13.31		
Fixed effects	**estimate**	***SE***	***t***	***P***
Intercept	80.33	11.94	6.73	**< 0.001**
Eggs (yes/no)	-13.68	16.87	-0.81	0.417
Feeding(yes/no)	43.46	15.67	2.77	**0.006**
Eggs x Feeding	-5.53	23.07	-0.24	0.810

Table S9. Statistical comparisons to assess the effect of *S. exigua* oviposition and feeding on transcript accumulation of selected genes in *S. dulcamara*. Welch's t-tests on qPCR data of (**a**) *anthocynidin synthase* (*ANS*), (**b**) *glutathione S-transferase* (*GST*), (**c**) *hydroxycinnamoyl CoA quinate transferase* (*HQT*) and (**d**) *major latex-like protein* (*MLP*). Transcripts were determined in RNA from leaf tissue of *S. dulcamara* plants that were either untreated (Control: C), previously oviposited (Eggs: E), fed by *S. exigua* larvae (Feeding: F) or fed and previously oviposited (Eggs + feeding: EF). Significant P-values are highlighted in bold.

**a) Welsh's t-tests on *ANS* transcript accumulation in the L1-leaf systemic to oviposition**			
Comparison	***t***	**df**	***P***
C-E	0.99	8.17	0.350
C-F	1.63	9.18	0.137
C-EF	-1.03	9.04	0.330
E-EF	-1.57	11.60	0.143
F-EF	2.12	11.99	0.055
**b) Welsh's t-tests on *GST* transcript accumulation in the L1-leaf systemic to oviposition**			
Comparison	***t***	**df**	***P***
C-E	1.16	8.18	0.280
C-F	2.87	9.15	**0.018**
C-EF	-0.35	10.31	0.735
E-EF	-1.28	10.45	0.228
F-EF	2.78	11.55	**0.017**
**c) Welsh's t-tests on *HQT* transcript accumulation in the L1-leaf systemic to oviposition**			
Comparison	***t***	**df**	***P***
C-E	0.76	8.61	0.499
C-F	2.85	8.90	**0.019**
C-EF	-0.97	11.94	0.350
E-EF	-12.96	8.30	0.230
F-EF	3.50	8.56	**0.007**
**d) Welsh's t-tests on *MLP* transcript accumulation in the L1-leaf systemic to oviposition**			
Comparison	***t***	**df**	***P***
C-E	-0.26	11.80	0.801
C-F	0.23	11.59	0.819
C-EF	-1.50	11.85	0.161
E-EF	-1.34	11.35	0.207
F-EF	1.55	11.93	0.139
